# The Ethnopharmacological Uses, Metabolite Diversity, and Bioactivity of *Rhaponticum uniflorum* (*Leuzea uniflora*): A Comprehensive Review

**DOI:** 10.3390/biom12111720

**Published:** 2022-11-20

**Authors:** Daniil N. Olennikov

**Affiliations:** Laboratory of Biomedical Research, Institute of General and Experimental Biology, Siberian Division, Russian Academy of Science, Sakh’yanovoy Street 6, 670047 Ulan-Ude, Russia; olennikovdn@mail.ru; Tel.: +7-902-160-06-27

**Keywords:** *Rhaponticum uniflorum*, Compositae (Asteraceae), ecdysteroids, flavonoids, thiophenes, HPLC, anti-inflammatory activity, neuroprotection

## Abstract

*Rhaponticum uniflorum* (L.) DC. (*syn*. *Leuzea uniflora* (L.) Holub) is a plant species of the Compositae (Asteraceae) family that is widely used in Asian traditional medicines in China, Siberia, and Mongolia as an anti-inflammatory and stimulant remedy. Currently, *R. uniflorum* is of scientific interest to chemists, biologists, and pharmacologists, and this review includes information from the scientific literature from 1991 to 2022. The study of the chemodiversity of *R. uniflorum* revealed the presence of 225 compounds, including sesquiterpenes, ecdysteroids, triterpenes, sterols, thiophenes, hydroxycinnamates, flavonoids, lignans, nucleosides and vitamins, alkanes, fatty acids, and carbohydrates. The most studied groups of substances are phenolics (76 compounds) and triterpenoids (69 compounds). Information on the methods of chromatographic analysis of selected compounds, as well as on the quantitative content of some components in various organs of *R. uniflorum*, is summarized in this work. It has been shown that the extracts and some compounds of *R. uniflorum* have a wide range of biological activities, including anti-inflammatory, antitumor, immunostimulatory, anxiolytic, stress-protective, actoprotective, antihypoxic, anabolic, hepatoprotective, inhibition of PPARγ receptors, anti-atherosclerotic, and hypolipidemic. Published research on the metabolites and bioactivity of *R. uniflorum* does not include clinical studies of extracts and pure compounds; therefore, an accurate study of this traditional medicinal plant is needed.

## 1. Introduction

*Rhaponticum* Vaill. is a small genus from the tribe Cynareae of the Asteraceae family that is distributed mainly in tropical and subtropical regions of Europe, Asia, and Africa. In total, more than 20 species belong to the genus and are distributed in a narrow strip in the Northern hemisphere from the Atlantic coast to the Pacific Ocean [1]. Close to *Rhaponticum* are the Mediterranean monotypic genus *Leuzea* and the small Asian genus *Stemmacantha*, which, combined, include approximately 10 species. Many species of *Rhaponticum* are of economic importance, and some have been introduced into cultivation as ornamental or medicinal plants. *R. carthamoides* (also known as Maral root) is widespread from Central Asia to Siberia and Xinjiang; it is a medicinal plant and a source of ecdysteroids; it is recommended as part of combination therapy for asthenia, physical and mental overwork, impotency, and during convalescence [2]. North African endemic species *R. acaule* is used as an aperitif, cholagogue, depurative, digestive, stomachic, and tonic in North and Central Tunisia [3]. Creeping knapweed or *R. repens* is a traditional medicine in Central Asia; it is applied as an emetic, antiepileptic, and anti-malaria remedy [4].

One-flowered leuzea or *Rhaponticum uniflorum* (L.) DC. (synonyms—*R. dauricum* Turcz., *R. monanthum* (Georgi) Worosch., *Centaurea monanthos* Georgi, *C. grandiflora* Pall., *C. membranaceae* Lam., *Serratula uniflora* Spreng., *Leuzea daurica* Bge., and *L. uniflora* (L.) Holub.) has received considerable attention in recent years. There are some scientific study reviews dedicated to *R. carthamoides* [2] and the genus *Rhaponticum* [5]; however, the issues of *R. uniflorum* are not fully covered. Therefore, the aim of this work is to summarize scientific information about *R. uniflorum* regarding the chemical composition of the herb and roots, as well as methods of analysis and biological activity.

**Figure 1:**
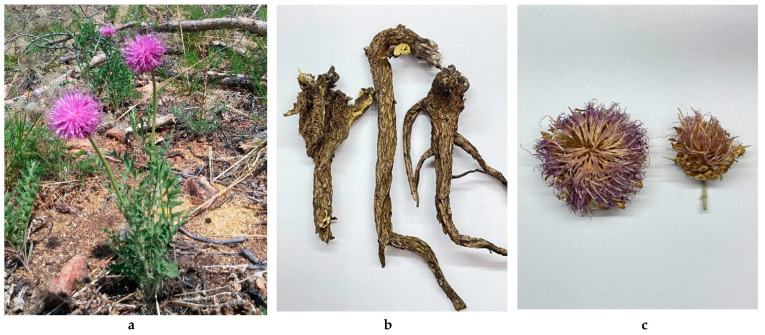
*Rhaponticum uniflorum* (L.) DC. (one-flowered leuzea) in its natural habitat (Republic Buryatia, Ivolginskii District, Kluchi vicinity, mountain slope; (**a**)), and dried roots (*qizhou loulu*; (**b**)) and flowers (*louluhua***,** *spyang-tser*; (**c**)).

Botanically, *R. uniflorum* is a low- or medium-height plant (20–60-cm tall) with straight, simple, felted stems [1,2]. Its leaves are rough on both sides, with adpressed cobwebby pubescence, pinnately divided into 8–12 pairs of dentate or entire obtuse lobes. The basal and lower leaves are petiolate, and the upper ones are sessile. Single inflorescences (3–5-cm wide) have outer and middle leaflets that are adpressed, leathery, light-brown, bare, broadly ovate, contain shiny appendages, and are split at the top into uneven lobes. Flower corolla is slightly funnel-shaped and has a coloration ranging from pale pink to red. The rhizome is thick, long, and vertical, with a loose, tuberous-fibrous surface and a few thin roots. Flowers are collected in late spring and early summer, and the roots are dug up in early autumn (Figure 1). In nature, *R. uniflorum* is scattered on meadow-steppe mountain slopes, along sandy riverbanks, and in the forests of Eastern Siberia and the Russian Far East, as well as in Northern Mongolia, Northeastern China, and Korea [6].

## 2. Review Strategy

To produce a relevant review, international databases (e.g., Scopus, Web of Science, PubMed, and Google Scholar) were used. Because most studies have been performed by Chinese and Russian scientists, national electronic resources (e.g., Chinese research databases (Wanfang and CNKI Journals) and the Russian scientific database (eLibrary)) were included in the search. These resources contain relevant articles that are not indexed by international databases. Only original papers written in English, Chinese, and Russian, and published in journals prior to October 2022, were considered. An exception was made for the ethnopharmacological data collected from books. The search keywords used included plant names (e.g., “*Rhaponticum uniflorum*”, “*Leuzea uniflora*”, “*Stemmacantha uniflora*”, “*Fornicium uniflorum*”) and metabolite names. The list of *R. uniflorum* compounds includes secondary metabolites mostly correlated with ethnopharmacological uses and bioactivities of the plant, and, for a more complete picture, information about primary metabolites is also mentioned in this manuscript.

## 3. Ethnopharmacology

Ethnopharmacological uses of roots, flowers, and the herb of *R. uniflorum* were found in Asian traditional medicines (Table 1). 

In traditional Chinese medicine, the roots of *R. uniflorum* (qizhou loulu) have been used as an anti-inflammatory, antipyretic, detoxifier, antitumor, and lactation agent [7], while flowers (louluhua) have the functions of relieving burning pain, clearing ‘heat’ (or ‘fire’), and as a detoxifying remedy [8]. In the Buryatia Republic, in addition to *R. uniflorum* [9], under the name spyang-tser, flowers of *R. carthamoides*, as well as the flowers and roots of *Carduus crispus*, Guirão ex Nyman, and *Cirsium esculentum* (Siev.) C.A.Mey., are used to treat stomach inflammations, gastroenteritis, pneumonia, bronchitis, and tuberculosis [10]. In Tibetan medicine, spyang-tser plants are prescribed for cleansing wounds and ulcers, indigestion, and other diseases of the stomach [11], lung diseases [12], and to treat skin diseases (boils, carbuncles), mastitis, and rheumatoid arthritis [13]. In Mongolian folk medicine, the *R. uniflorum* herb (khonkhor zul, spyang-tser, spyang-tser-dmar-po) is used as a water decoction, as an anti-inflammatory remedy, and to increase the vitality of the body [14]. In Korea, young buds of *R. uniflorum* are a food product, and the roots (nuro) are used to treat chronic gastritis as an anti-inflammatory, detoxifier, antipyretic, and analgesic agent [15]. Roots and flowers of *R. uniflorum* are traditional Chinese remedies recorded in the Chinese pharmacopeia and the “Drug Standard of the Ministry of Public Health of the People’s Republic of China” [16].

**Table 1:** Traditional medical uses of *R. uniflorum*.

Plant Part	Locality	Traditional Use	Ref.
Roots	China	Anti-inflammatory, antipyretic, detoxifier, antitumor, lactation remedy	[7]
Flowers	China	Relieving burning pain, clearing heat, detoxifying remedy	[9]
	Buryatia	Anti-inflammatory remedy at stomach deseases, gastroenteritis, pneumonia, bronchitis, tuberculosis	[10,11]
	Tibet	Remedy for cleansing wounds and ulcers, indigestion, stomach and lung diseases, to treat skin diseases (boils, carbuncles), mastitis, rheumatoid arthritis	[12,13,14]
Herb	Mongolia	Anti-inflammatory remedy, increasing the vitality of the body	[15]
Buds	Korea	Anti-inflammatory, detoxifier, antipyretic, and analgesic agent	[8]

## 4. Metabolite Diversity

More than 200 compounds (**1**–**225**) have been detected in various organs of *R. uniflorum*, including sesquiterpenes (**1**–**14**), diterpenes (**15**–**17**), triterpenes (**18**–**86**), thiophenes (**87**–**98**), hydroxycinnamates (**99**–**108**), flavonoids (**109**–**162**), lignans (**163**–**170**), various phenolics (**171**–**174**), amino acids (**175**–**187**), nucleosides and vitamins (**188**–**195**), alkanes (**196**–**199**), fatty acids (**200**–**217**), and carbohydrates (**218**–**225**) (Table 2).

### 4.1. Sesquiterpenes

**Figure 2:**
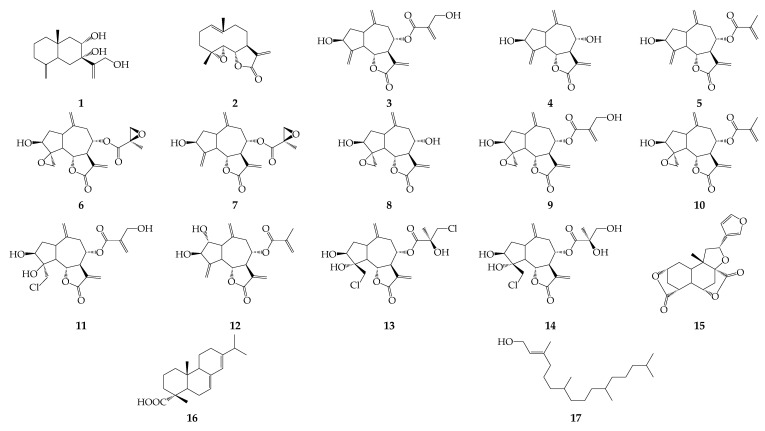
Sesquiterpenes **1**–**14** and diterpenes **15**–**17**.

Fourteen sesquiterpenes (**1**–**14**) have been identified in *R. uniflorum*, including eudesmane **1**, germacranolide **2**, and guaianes **3**–**14** [17,18,19,20] (Figure 2). Rhaponticol {7α,8α,12-trihydroxy-eudesma-4(15)-11(13)-diene, **1**}, isolated from roots of *R. uniflorum* [17], is the only eudesmane found in the *Rhaponticum* genus, and it is non-typical for the Rhaponticum group (Centaureinae subtribe). This sesquiterpene type is characteristic of other members of the tribe, including the genus *Centaurea* (Centaurea group) and, less commonly, for the Mediterranean species *Cheirolophus* and *Phonus* (Carthamus group) [20]. 

**Table 2:** Compounds **1**–**225** found in *R. uniflorum*.

No	Compound	Formula	MW *	Herb	Leaves	Flowers	Seeds	Roots
	Sesquiterpenes							
**1**	Rhaponticol	C_15_H_24_O_3_	252					[17]
**2**	Parthenolide	C_15_H_20_O_3_	248			[9]		
**3**	Cynaropicrin	C_19_H_22_O_6_	364	[18]	[19]	[19]	[19]	[19]
**4**	Cynaropicrin, desacyl-	C_15_H_18_O_4_	262		[19]			
**5**	Cynaropicrin, 4′-deoxy- (aguerin B)	C_19_H_22_O_5_	330	[18]	[19]	[19]	[19]	[19]
**6**	Repin	C_19_H_22_O_7_	362		[19]			
**7**	Repin, 15-desoxy- (salograviolide C)	C_17_H_20_O_6_	320	[18]	[19]			[19]
**8**	Repin, 8-desacyl-	C_15_H_18_O_5_	278		[19]			
**9**	Janerin	C_19_H_22_O_7_	362		[19]			
**10**	Janerin, 19-desoxy-	C_19_H_22_O_6_	346		[19]			
**11**	Janerin, chloro-	C_19_H_23_ClO_7_	398.5		[19]			
**12**	Repdiolide	C_19_H_22_O_6_	346		[19]			
**13**	Chlorohyssopifolin A (centaurepensin, hyrcanin)	C_19_H_24_Cl_2_O_7_	435		[19]			[20]
**14**	Chlorohyssopifolin E	C_19_H_25_ClO_8_	416		[19]			
	Diterpenes							
**15**	Diosbulbin B	C_19_H_20_O_6_	344					[21]
**16**	Abietic acid	C_20_H_30_O_2_	302			[9]		
**17**	Phytol	C_20_H_40_O	296	[6]				
	Triterpenes							
**18**	Ajugasteron C	C_27_H_44_O_7_	480	[6]	[22]			[23,24,25]
**19**	Ajugasteron C 20,22-acetonide	C_30_H_48_O_7_	520		[22]			[23,24,25]
**20**	Ajugasteron C 2,3;20,22-diacetonide	C_33_H_52_O_7_	560		[22]			[23,24,26]
**21**	5-Deoxycaladasterone (dacryhainansterone)	C_27_H_42_O_6_	462		[22]	[27]		
**22**	5-Deoxycaladasterone (dacryhainansterone) 20,22-acetonide	C_30_H_46_O_6_	502		[22]	[27]		[16,17]
**23**	2-Deoxyecdysone	C_27_H_44_O_5_	448		[22]			
**24**	25-Deoxyecdysone	C_27_H_44_O_5_	448		[22]			
**25**	2-Deoxy-20-hydroxyecdysone	C_27_H_44_O_6_	464	[28]	[22]		[29]	[28]
**26**	Ecdysone	C_27_H_44_O_6_	464	[6]				
**27**	11α-Hydroxyecdysone	C_27_H_44_O_7_	480					[23]
**28**	20-Hydroxyecdysone	C_27_H_44_O_7_	480	[7,28,30]	[22]	[27]	[29]	[7,23,24,25,31,32]
**29**	20-Hydroxyecdysone 2-*O*-acetate	C_29_H_46_O_8_	522		[22]			
**30**	20-Hydroxyecdysone 3-*O*-acetate	C_29_H_46_O_8_	522		[22]	[27]		
**31**	20-Hydroxyecdysone 25-*O*-acetate (viticosterone E)	C_29_H_46_O_8_	522	[6]				
**32**	20-Hydroxyecdysone 20,22-acetonide	C_30_H_48_O_7_	520	[6]	[22]	[27]		
**33**	20-Hydroxyecdysone 2,3;20,22-diacetonide	C_33_H_52_O_7_	560		[22]			
**34**	20-Hydroxyecdysone 3-*O*-glucoside	C_33_H_54_O_12_	642					[6]
**35**	20-Hydroxyecdysone 25-*O*-glucoside	C_33_H_54_O_12_	642					[6]
**36**	20-Hydroxyecdysone 2-*O*-cinnamate	C_36_H_50_O_8_	610		[33]			
**37**	29-Hydroxy-24(28)-dehydromakisterone C	C_29_H_46_O_8_	522		[22]			
**38**	Inokosterone (callinecdysone A)	C_27_H_44_O_7_	480		[22]	[27]		
**39**	Inokosterone 20,22-acetonide	C_30_H_48_O_7_	520		[22]			
**40**	Inokosterone 20,22-acetonide 25-*O*-acetate	C_32_H_50_O_8_	562		[22]			
**41**	Integristerone A	C_27_H_44_O_8_	496	[28]	[22]			[28]
**42**	Integristerone A 20,22-acetonide	C_30_H_48_O_8_	536		[22]	[27]		
**43**	Makisterone C (podecdysone A, lemmasterone)	C_29_H_48_O_7_	508		[22]			
**44**	Makisterone C 20,22-acetonide	C_32_H_52_O_7_	548		[27]	[27]		
**45**	Polypodine B	C_27_H_44_O_8_	496		[22]			
**46**	Polypodine B 20,22-acetonide	C_30_H_48_O_8_	536		[27]			
**47**	Polypodine B 2-*O*-cinnamate	C_36_H_50_O_9_	626		[33]			
**48**	Ponasterone A	C_27_H_44_O_6_	464		[22]			
**49**	Rapisterone C	C_29_H_48_O_7_	508					[23]
**50**	Rhapontisterone (punisterone)	C_27_H_44_O_8_	496	[7]	[22]			[7,23,31,32]
**51**	Rhapontisterone R_1_	C_29_H_42_O_9_	534					[32]
**52**	Rubrosterone	C_19_H_26_O_5_	334	[6]				
**53**	Turkesterone	C_27_H_44_O_8_	496	[7,30]	[22]			[7,31]
**54**	Turkesterone 20,22-acetonide	C_30_H_48_O_8_	536		[22]			
**55**	Turkesterone 2-*O*-cinnamate	C_36_H_50_O_9_	626		[33]			
**56**	Uniflorsterone	C_27_H_44_O_7_	480					[34]
**57**	Roburic acid	C_30_H_48_O_2_	440			[9]		
**58**	Urs-12-en-3-one (α-amyrenone)	C_30_H_48_O	424					[35]
**59**	Urs-12-en-3β-ol (α-amyrin)	C_30_H_50_O	426	[35]				[35]
**60**	3-Oxo-urs-12-en-24-oic acid methyl ester	C_31_H_48_O_3_	468	[35]				
**61**	3β-Hydroxy-urs-12-en-28-oic acid (ursolic acid)	C_30_H_48_O_3_	456	[35]				[25,36,37]
**62**	3β-Hydroxy-urs-12,18(19)-dien-28-oic acid 28-*O*-glucoside	C_36_H_56_O_8_	616					[25]
**63**	3β-Hydroxy-urs-12,18(19)-dien-28-oic acid 3-*O*-arabinoside-28-*O*-glucoside	C_41_H_64_O_12_	748					[25]
**64**	3β-Hydroxy-urs-12,18(19)-dien-28-oic acid 3,28-di-*O*-glucoside	C_42_H_66_O_13_	778					[38]
**65**	3β-Hydroxy-urs-9(11),12-dien-28-oic acid 3-*O*-arabinoside-28-*O*-glucoside (unifloroside)	C_41_H_64_O_12_	748					[39]
**66**	3β-Hydroxy-urs-12,19(29)-dien-28-oic acid 28-*O*-glucoside	C_36_H_56_O_8_	616					[25]
**67**	3β-Hydroxy-urs-12,19(29)-dien-28-oic acid 3,28-di-*O*-glucoside	C_42_H_66_O_13_	778					[38]
**68**	3β,19α-Dihydroxy-urs-12-en-28-oic acid (pomolic acid)	C_30_H_48_O_4_	472					[25,40]
**69**	Pomolic acid 28-*O*-glucoside	C_36_H_58_O_9_	634					[25,39]
**70**	Pomolic acid 3-*O*-arabinoside-28-*O*-glucoside (ziyuglycoside I)	C_41_H_66_O_13_	766					[25,39]
**71**	Pomolic acid 3-*O*-arabinoside (ziyuglycoside II)	C_35_H_56_O_8_	604					[25,39]
**72**	3-Oxo-19α-hydroxy-urs-12-en-28-oic acid	C_30_H_46_O_4_	470					[25,36,40]
**73**	2α,3β,19α-Trihydroxy-urs-12-en-28-oic acid (tormentic acid)	C_30_H_48_O_5_	488					[36]
**74**	Tormentic acid 28-*O*-glucoside (rosamutin, rosamultin)	C_36_H_58_O_10_	650					[25,39]
**75**	2α,3β,19α-Trihydroxy-urs-12-en-23,28-dioic acid 28-*O*-glucoside (sauvissimoside R_1_)	C_36_H_56_O_12_	680					[25,39]
**76**	2α,3α,19α-Trihydroxy-urs-12-en-28-oic acid	C_30_H_48_O_5_	488					[18,29]
**77**	2α,3α,19α,25-Tetrahydroxy-urs-12-en-28-oic acid	C_30_H_48_O_6_	504					[40]
**78**	2α,3α,19α,25-Tetrahydroxy-urs-12-en-23,28-dioic acid	C_30_H_46_O_8_	534					[25]
**79**	Olean-12-en-3β-ol (β-amyrin)	C_30_H_50_O	426	[35]				[35]
**80**	3β-Hydroxy-olean-12-en-28-oic acid (oleanolic acid)	C_30_H_48_O_3_	456					[41]
**81**	2α,3β,19α-Trihydroxy-olean-12-en-28-oic acid (arjunic acid)	C_30_H_48_O_5_	488					[36]
**82**	β-Sitosterol	C_29_H_50_O	414	[35]				[40,41]
**83**	β-Sitosterol 28-*O*-glucoside (daucosterol)	C_35_H_60_O_6_	576					[25]
**84**	Stigmasterol	C_29_H_48_O	412	[35]				[41]
**85**	Stigmastan-3,5-diene	C_29_H_48_	396	[35]				[35]
**86**	Stigmast-4-en-3-on	C_29_H_48_O	412					[35]
	Thiophenes							
**87**	Arctinal	C_12_H_8_OS_2_	232					[17,41]
**88**	Arctinone b	C_13_H_10_OS_2_	246					[17,41,42]
**89**	Arctinone b, 7-chloro-	C_13_H_9_ClOS_2_	280.5					[41,42]
**90**	Arctinol b	C_13_H_12_O_2_S_2_	264					[17]
**91**	Arctic acid	C_12_H_8_O_2_S_2_	248					[17,25,40]
**92**	2,2′-Dithiophene, 5-methoxy-	C_9_H_8_OS_2_	196					[41]
**93**	2,2′-Dithiophene, 5-methoxy-5′-(1-propynyl)-	C_12_H_10_OS_2_	234					[41]
**94**	2,2′-Dithiophene, 5-(4-acetoxy-1-butynyl)-	C_14_H_12_O_2_S_2_	276					[41]
**95**	Rhaponthienylenol	C_13_H_14_O_3_S_2_	282					[6]
**96**	Rhapontiynethiophene A	C_11_H_7_ClS_2_	238.5					[42]
**97**	Rhapontiynethiophene B	C_13_H_10_O_2_S	230					[42]
**98**	Thiophene, 2-(pentadiynyl-1,3)-5-(3,4-dihydroxy-butynyl-1)-	C_13_H_10_O_2_S	230					[17]
	Hydroxycinnamates							
**99**	Cinnamic acid	C_9_H_8_O_2_	148			[9]		
**100**	Cinnamaldehyde	C_9_H_8_O	132			[9]		
**101**	4-*O*-Caffeoylquinic acid	C_16_H_18_O_9_	354	[43]			[29]	
**102**	5-*O*-Caffeoylquinic acid	C_16_H_18_O_9_	354	[43]		[9]	[29]	
**103**	1,3-Di-*O*-caffeoylquinic acid	C_25_H_24_O_12_	516	[43]				
**104**	1,5-Di-*O*-caffeoylquinic acid	C_25_H_24_O_12_	516	[43]				
**105**	3,4-Di-*O*-caffeoylquinic acid	C_25_H_24_O_12_	516	[43]			[29]	
**106**	3,5-Di-*O*-caffeoylquinic acid	C_25_H_24_O_12_	516	[30]		[9]	[29]	
**107**	4,5-Di-*O*-caffeoylquinic acid	C_25_H_24_O_12_	516				[29]	
**108**	Isoferuoyl serotonin	C_20_H_20_N_2_O_4_	352				[29]	
	Flavonoids							
**109**	Apigenin	C_15_H_10_O_5_	270		[33]	[16]		
**110**	Apigenin 7-*O*-glucoside (cosmosiin)	C_21_H_20_O_10_	432		[33]	[16]		
**111**	Apigenin 7-*O*-glucuronide	C_21_H_18_O_11_	446		[33]	[16]		
**112**	Apigenin 6-*C*-glucoside (isovitexin)	C_21_H_20_O_10_	432		[33]			
**113**	Apigenin 8-*C*-glucoside (vitexin)	C_21_H_20_O_10_	432		[33]	[9]		
**114**	Apigenin 6,8-di-*C*-glucoside (vicenin-2)	C_27_H_30_O_15_	594			[16]		
**115**	6-Methoxyapigenin (hispidulin)	C_16_H_12_O_6_	300		[33]			
**116**	Luteolin	C_15_H_10_O_5_	286			[16]	[29]	
**117**	Luteolin 7-*O*-glucoside (cynaroside)	C_21_H_20_O_11_	448		[33]			
**118**	Luteolin 7-*O*-(6″-*O*-cinnamoyl)-glucoside	C_30_H_26_O_12_	578		[33]		[29]	
**119**	Luteolin 7-*O*-(2″-*O*-caffeoyl)-glucoside (rhaunoside G)	C_30_H_26_O_14_	610		[33]			
**120**	Luteolin 7-*O*-(6″-*O*-caffeoyl)-glucoside	C_30_H_26_O_14_	610		[33]			
**121**	Luteolin 7-*O*-glucuronide	C_21_H_18_O_12_	462		[33]			
**122**	Luteolin 7-*O*-rutinoside (scolymoside)	C_27_H_30_O_15_	594		[33]			
**123**	Luteolin 3′-*O*-glucoside (dracocephaloside)	C_21_H_20_O_11_	448		[33]			
**124**	Luteolin 4′-*O*-glucoside	C_21_H_20_O_11_	448		[33]			
**125**	Luteolin 6-*C*-glucoside (isoorientin)	C_21_H_20_O_11_	448		[33]			
**126**	Luteolin 8-*C*-glucoside (orientin)	C_21_H_20_O_11_	448		[33]			
**127**	Luteolin 6,8-di-*C*-glucoside (lucenin-2)	C_27_H_30_O_16_	610		[33]			
**128**	3′-Methoxyluteolin (chrysoeriol)	C_16_H_12_O_6_	300		[33]	[30]		
**129**	6-Hydroxyluteolin	C_15_H_10_O_6_	302		[33]			
**130**	6-Hydroxyluteolin 7-*O*-glucoside	C_21_H_20_O_12_	464		[33]		[29]	
**131**	6-Hydroxyluteolin 7-*O*-(6″-*O*-cinnamoyl)-glucoside (rhaunoside B)	C_30_H_26_O_13_	594		[33]		[29]	
**132**	6-Hydroxyluteolin 7-*O*-(2″-*O*-caffeoyl)-glucoside (rhaunoside A)	C_30_H_26_O_15_	626					
**133**	6-Hydroxyluteolin 7-*O*-(6″-*O*-caffeoyl)-glucoside (spicoside A)	C_30_H_26_O_15_	626		[33]			
**134**	6-Hydroxyluteolin 7-*O*-rutinoside	C_27_H_30_O_16_	610		[33]			
**135**	6-Hydroxyluteolin 4′-*O*-glucoside (rhaunoside C)	C_21_H_20_O_12_	464		[33]			
**136**	6-Methoxyluteolin (nepetin)	C_16_H_12_O_7_	316		[33]			
**137**	6-Methoxyluteolin 7-*O*-glucoside (nepitrin)	C_22_H_22_O_12_	478		[33]			
**138**	6-Methoxyluteolin 7-*O*-(6″-*O*-cinnamoyl)-glucoside (rhaunoside E)	C_31_H_28_O_13_	608		[33]			
**139**	6-Methoxyluteolin 7-*O*-(6″-*O*-caffeoyl)-glucoside (rhaunoside D)	C_31_H_28_O_15_	640		[33]			
**140**	6-Methoxyluteolin 7-*O*-rutinoside	C_28_H_32_O_16_	624		[33]			
**141**	6-Methoxyluteolin 3′-*O*-glucoside (rhaunoside F)	C_22_H_22_O_12_	478		[33]			
**142**	6-Methoxyluteolin 4′-*O*-glucoside	C_22_H_22_O_12_	478		[33]			
**143**	6,8-Dihydroxyluteolin 7-*O*-glucoside (zeravschanoside)	C_21_H_20_O_13_	480		[33]			
**144**	5,6,7,4′-Tetrahydroxy-3′-methoxyflavone (nodifloretin)	C_16_H_12_O_7_	316		[33]			
**145**	5,6,7,3′-Tetrahydroxy-4′-methoxyflavone	C_16_H_12_O_7_	316		[33]			
**146**	Kaempferol	C_15_H_10_O_6_	286			[30]		
**147**	Kaempferol 3-*O*-rhamnoside (quercitrin)	C_21_H_20_O_11_	448			[30]		
**148**	6-Hydroxykaempferol	C_15_H_10_O_7_	302		[33]			
**149**	6-Hydroxykaempferol 7-*O*-glucoside	C_21_H_20_O_12_	464		[33]			
**150**	6-Hydroxykaempferol 7-*O*-(6″-*O*-caffeoyl)-glucoside	C_30_H_26_O_15_	626		[33]			
**151**	6-Methoxykaempferol 7-*O*-glucoside	C_22_H_22_O_12_	478		[33]			
**152**	Quercetin	C_15_H_10_O_7_	302			[30]		
**153**	Quercetin 3-*O*-rhamnoside (quercitrin)	C_21_H_20_O_11_	448			[9]		
**154**	Quercetin 3-*O*-glucoside (isoquercitrin)	C_21_H_20_O_12_	464			[9]		
**155**	Quercetin 3-*O*-rutinoside (rutin)	C_27_H_30_O_16_	610			[9]		
**156**	6-Hydroxyquercetin (quercetagetin)	C_15_H_10_O_8_	318		[33]			
**157**	6-Hydroxyquercetin 7-*O*-glucoside (quercetagitrin)	C_21_H_20_O_13_	480		[33]			
**158**	6-Hydroxyquercetin 7-*O*-(6″-*O*-caffeoyl)-glucoside	C_30_H_26_O_16_	642		[33]			
**159**	6-Methoxyquercetin 7-*O*-glucoside (patulitrin)	C_22_H_22_O_13_	494		[33]			
**160**	3′-Methoxyquercetin (isorhamnetin)	C_16_H_12_O_6_	300		[33]	[9]		
**161**	4′-Methoxyquercetin (diosmetin)	C_16_H_12_O_6_	300			[30]		
**162**	Catechin	C_15_H_14_O_6_	190					[25]
	Lignans							
**163**	Hemislin B					[30]		
**164**	Hemislin B *O*-glucoside					[30]		
**165**	Arctigenin	C_21_H_24_O_6_	372			[9]		
**166**	Arctigenin *O*-glucoside (arctiin)	C_27_H_34_O_11_	534			[9]		
**167**	Carthamogenin	C_21_H_22_O_6_	370				[29]	
**168**	Carthamoside	C_27_H_32_O_11_	532				[29]	
**169**	6″-*O*-Acetyl carthamoside	C_29_H_34_O_12_	574				[29]	
**170**	Tracheloside	C_27_H_34_O_12_	550				[29]	
	Other phenolics							
**171**	3,5-Dimethoxy-4-hydroxybenzaldehyde (syringaldehyde)	C_9_H_10_O_4_	182			[9]		
**172**	3,3′,4-Tri-*O*-methyl-ellagic acid	C_17_H_12_O_8_	344					[25]
**173**	Coumarin	C_9_H_6_O_2_	146			[9]		
**174**	Ligustilide	C_12_H_14_O_2_	190			[9]		
	Amino acids							
**175**	Alanin	C_3_H_7_NO_2_	89	[44]				[44]
**176**	Arginin	C_6_H_14_N_4_O_2_	174	[44]				[44]
**177**	Glycine	C_2_H_5_NO_2_	75	[44]				[44]
**178**	Histidin	C_6_H_9_N_3_O_2_	155					[44]
**179**	Lysine	C_6_H_14_N_2_O_2_	146	[44]				[44]
**180**	Leucin	C_6_H_13_NO_2_	131	[44]				
**181**	Methionine	C_5_H_11_NO_2_S	149					[44]
**182**	Phenylalanine	C_9_H_11_NO_2_	165	[44]				[44]
**183**	Proline	C_5_H_9_NO_2_	115	[44]				[44]
**184**	Serine	C_3_H_7_NO_3_	105	[44]				[44]
**185**	Tyrosine	C_9_H_11_NO_3_	181	[44]				[44]
**186**	Threonine	C_4_H_9_NO_3_	119	[44]				[44]
**187**	Valin	C_5_H_11_NO_2_	117					[44]
	Nucleosides and vitamins							
**188**	Cordycepin (3′-deoxyadenosine)	C_10_H_13_N_5_O_3_	251			[9]		
**189**	Thiamine (vitamin B_1_)	C_12_H_17_N_4_OS^+^	265	[45]				[45]
**190**	Riboflavine (vitamin B_2_)	C_17_H_20_N_4_O_6_	376	[45]				[45]
**191**	Pantothenic acid (vitamin B_5_)	C_9_H_17_NO_5_	219	[45]				[45]
**192**	Nicotinic acid (niacin, vitamin B_3_)	C_6_H_5_NO_2_	123	[45]				[45]
**193**	Nicotinamide	C_6_H_6_N_2_O	122			[9]		
**194**	Pyridoxine (vitamin B_6_)	C_8_H_11_NO_3_	169	[45]				[45]
**195**	Folic acid (vitamin B_9_)	C_19_H_19_N_7_O_6_	441					[45]
	Alkanes							
**196**	Pentacosane	C_25_H_52_	352	[35]				
**197**	Heptacosane	C_27_H_56_	380	[35]				
**198**	Octacosane	C_28_H_58_	394	[35]				
**199**	Nonacosane	C_29_H_60_	408	[35]				
	Fatty acids							
**200**	Tetradecanoic acid (myristic acid; 14:0)	C_14_H_28_O_2_	228	[35]				[35]
**201**	Pentadecanoic acid (15:0)	C_15_H_30_O_2_	242	[35]				[35]
**202**	Hexadecanoic acid (palmitic acid; 16:0)	C_16_H_32_O_2_	256	[35]				[35]
**203**	Heptadecanoic acid (margaric acid; 17:0)	C_17_H_34_O_2_	270	[35]				[35]
**204**	Octadecanoic acid (stearic acid; 18:0)	C_18_H_36_O_2_	284	[35]				[35]
**205**	Icosanoic acid (arachic acid; 20:0)	C_20_H_40_O_2_	312	[35]				[35]
**206**	Heneicosanoic acid (21:0)	C_21_H_42_O_2_	326	[35]				
**207**	Docosanoic acid (behenic acid; 22:0)	C_22_H_44_O_2_	340	[35]				[35]
**208**	Tricosanoic acid (23:0)	C_23_H_46_O_2_	354	[35]				[35]
**209**	Tetracosanoic acid (lignoceric acid; 24:0)	C_24_H_48_O_2_	368	[35]				[35]
**210**	Pentacosanoic acid (25:0)	C_25_H_50_O_2_	382	[35]				[35]
**211**	Hexacosanoic acid (cerotic acid; 26:0)	C_26_H_52_O_2_	396	[35]				
**212**	Octacosanoic acid (montanic acid; 28:0)	C_28_H_56_O_2_	424	[35]				
**213**	Triacontanoic acid (melissic acid; 30:0)	C_30_H_60_O_2_	452	[35]				
**214**	Hexadec-7-enoic acid (16:1*n*9)	C_16_H_30_O_2_	254	[35]				[35]
**215**	Octadec-9-enoic acid (oleic acid; 18:1*n*9)	C_18_H_34_O_2_	282					[35]
**216**	Octadeca-9,12-dienoic acid (linoleic acid; 18:2*n*6)	C_18_H_32_O_2_	280	[35]				[35]
**217**	Octadeca-9,12,15-trienoic acid (linolenic acid; 18:3*n*3)	C_18_H_30_O_2_	278	[35]		[9]		[35]
	Carbohydrates							
**218**	Glucose	C_6_H_12_O_6_	180		[46]	[46]	[46]	[46]
**219**	Fructose	C_6_H_12_O_6_	180		[46]	[46]	[46]	[46]
**220**	Sucrose	C_12_H_22_O_11_	342		[46]	[46]	[46]	[46]
**221**	Kestose (1^F^-β-fructofuranosyl sucrose)	C_18_H_32_O_16_	504		[46]			[46]
**222**	Nystose (di-(1^F^-β-fructofuranosyl) sucrose)	C_24_H_42_O_21_	666		[46]			[46]
**223**	1^F^-β-Fructofuranosyl nystose	C_30_H_52_O_26_	828		[46]			[46]
**224**	Di-(1^F^-β-fructofuranosyl) nystose	C_36_H_62_O_31_	990		[46]			[46]
**225**	Tri-(1^F^-β-fructofuranosyl) nystose	C_42_H_72_O_36_	1152		[46]			[46]

* MW—Molecular weight.

Parthenolide (**2**), a typical feverfew component, has been found in *Centaurea* and *Stizolophus* genera [20], but it is the only germacranolide in the Rhaponticum group. Unlike eudesmanes and germacranolides, guaianes are widely distributed in *Rhaponticum* species, especially cynaropicrine (**3**), and are identified in *R. uniflorum* [18] and in *R. carthamoides* (Willd.) Iljin, *R. exaltatum* (Willk.) Greuter, *R. pulchrum* Fisch. & C.A.Mey., *R. scariosum* subsp. *Rhaponticum* (L.) Greuter, and *R. serratuloides* (Georgi) Bobrov [20]. Structurally similar to **3**, sesquiterpenes **4**–**12** have been isolated from the herb and roots of *R. uniflorum* [18,19], as well as two chlorinated sesquiterpenes, i.e., chlorohyssopifolins A (**13**) and E (**14**) [19,20].

### 4.2. Diterpenes

The member of furanoid norditerpenes diosbulbin B (**15**) was found in *R. uniflorum* roots (Figure 2) [21]. This compound, first isolated from *Dioscorea bulbifera* L. [47], is a hepatotoxic agent that causes oxidative damage to hepatocyte membranes [48]. Additionally, abietane diterpenoid abietic acid (**16**) and acyclic diterpene alcohol phytol (**17**) have been detected in the flowers and herb of *R. uniflorum*.

### 4.3. Triterpenes

**Figure 3:**
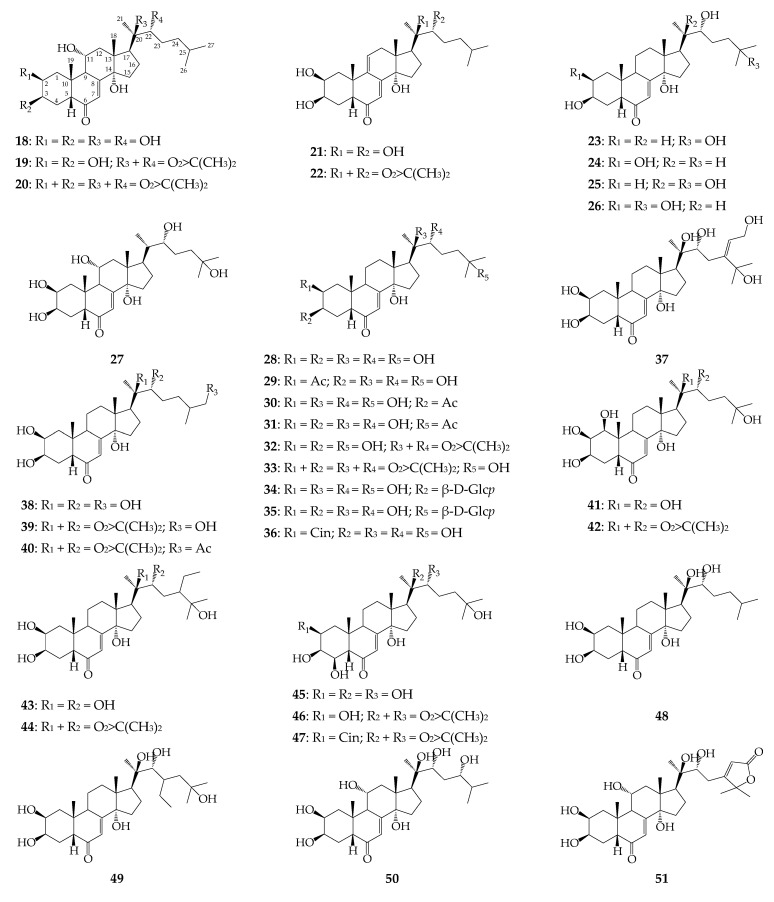

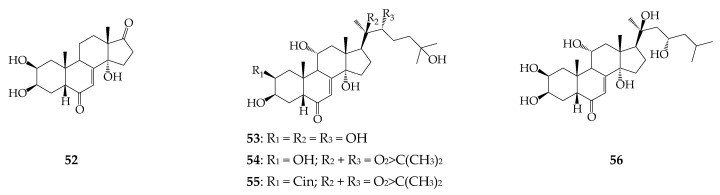
Ecdysteroids **18**–**56**. Ac–acetyl; Cin–cinnamoyl; β-D-Glc*p*–β-D-glucopyranose.

Various types of triterpenes were found in *R. uniflorum*, including ecdysteroids, triterpene acids, alcohols, ketones, and sterols. Ecdysteroids were first discovered in *R. uniflorum* in the early 1990s [31]. Since then, 39 compounds (**18**–**56**) of this group have been identified in the plant, of which 33 are in the herb (**18**–**26**, **28**–**33**, **36**–**48**, **50**, **52**–**55**) and 15 in the roots (**18**–**20**, **22**, **25**, **27**, **28**, **34**, **35**, **41**, **49**–**51**, **53**, **56**) (Figure 3). Almost all compounds contain a full side chain, except rubosterone (**16**). The number of hydroxyl groups in ecdysteroid structures can be 3 (**52**), 4 (**23**, **24**), 5 (**21**, **22**, **25**, **26**, **48**), 6 (**18**–**20**, **27**–**36**, **38**–**40**, **43**, **44**, **49**, **51**, **56**), and 7 (**37**, **41**, **42**, **45**–**47**, **50**, **53**–**55**), indicating the dominance of polyhydroxy compounds. The most common derivatives are 20-hydroxyedysone (**28**–**36**), ajugasterone C (**18**–**20**), inokosterone (**38**–**40**), polypodine B (**45**–**47**), and turkesterone (**53**–**55**). For individual components, acetates (**29**–**31**), acetonides (**19**, **22**, **32**, **39**, **42**, **44**, **46**, **54**), diacetonides (**20**, **33**), and acetonide-acetates (**40**) can be formed. Glycosides are a rare group of derivatives for *R. uniflorum* because only two compounds (**22** and **23**) have been identified in the roots of this species [6]. Ecdysteroids cinnamoyl esters **36**, **47**, and **55** found in the leaves of the plant deserve special attention [33]. Previously known compounds (**36** and **47**) were isolated only from the fern *Dacrydium intermedium* Kirk (*Lepidothamnus intermedius* (Kirk) Quinn, Podocarpaceae) [49,50]. The unusual structural compounds include rapontisteron R_1_ (**51**) (which contains a furan ring in the side chain [32]) and uniflorsterone (**56**) (which contains a hydroxyl group in the atom C-23 [34]). 

Comparing the chemodiversity of the ecdysteroids in *R. uniflorum* with that of the more-studied species *R. carthamoides* (in which more than 50 compounds of this class have been identified so far [20]), it can be assumed that there are many more compounds in the composition of the steroid metabolome of *R. uniflorum*.

**Figure 4:**
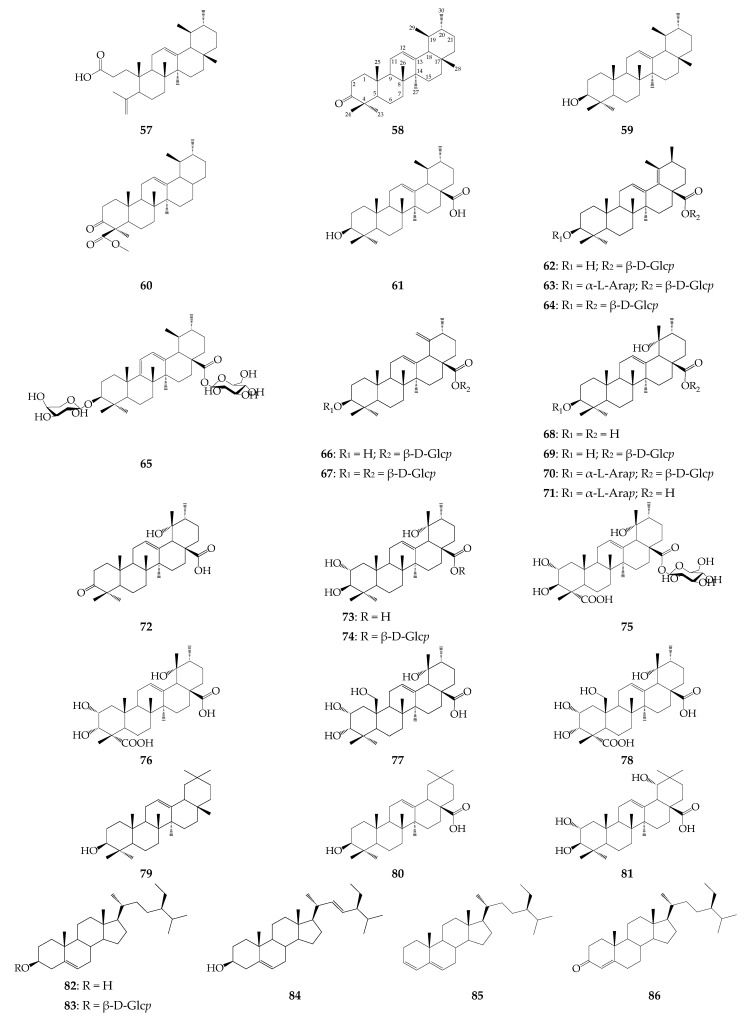
Triterpenes **57**–**86**. A-L-Ara*p*–α-L-arabinopyranose; β-D-Glc*p*–β-D-glucopyranose.

Different organs of *R. uniflorum* are the sources of 25 non-ecdysteroid triterpenoids (**57**–**81**), including 23 compounds isolated from the roots and five components detected in the herb (**57**, **59**–**61**, **79**) (Figure 4). The only tetracyclic triterpene roburic acid (**57**), typical for *Gentiana* roots [51], was detected in the flowers of *R. uniflorum* [9]. The remaining compounds (**58**–**81**) were pentacyclic triterpenes. Ursans are the dominant structural type of *R. uniflorum* triterpenes (21 compounds), as opposed to oleanans, represented by fewer components (3 compounds). Triterpenoids of *R. uniflorum* can contain unsaturated bonds at C_9_–C_11_, C_12_–C_13_, C_18_–C_19_, C_19_–C_29_, hydroxyl groups at C_2_, C_3_, C_19_, and C_25_ and carboxyl groups at C_23_ and C_28_. Eleven compounds have been identified as mono- and di-glycosides, including fragments of β-D-glucose and/or α-L-arabinose at C_3_ and/or C_28_. Two alcohols, α- (**59**) and β-amyrins (**79**) [35], as well as two acids, 3-oxo-ursus-12-en-24-oic acid (as methyl ether, **60**) [35] and ursolic acid (**61**) [30], have been detected in the *R. uniflorum* herb. Triterpenoids of *R. uniflorum* roots are notable for their large structural diversity of the primary ursan skeleton, as well as their ability to form glycosides identified only in this part of the plant. The basic triterpene aglycones are 3β-hydroxy-urs-12,18(19)-dien-28-oic acid as glycosides **62**–**64** [25,39], 3β-hydroxy-urs-12,19(29)-dien-28-oic acid as glycosides **66** and **67** [25,39], pomolic acid (3β,19α-dihydroxy-urs-12-en-28-oic acid, **68**) [25,41] and tormentic acid (2α,3β,19α-trihydroxy-urs-12-en-28-oic acid, **73**) [36]. Of note, the 3β-hydroxy functional group is typical for *R. uniflorum* triterpenoids, except in three compounds with a 3α-hydroxy functional group, including **76** [25,41], **77** [41], and **76** [25], isolated from the roots of *R. uniflorum* growing in China. A few oleanan derivatives include β-amyrin (**79**) [35], oleanolic acid (**80**) [40], and arjunic acid (**81**) [36]. Five stigmastane derivatives have been found in the *R. uniflorum* herb and roots, including β-sitosterol (**82**) and its glucosides daucosterol (**83**) [25,35,40,41], stigmasterol (**84**) [35,41], stigmastan-3,5-diene (**85**) [35], and stigmast-4-en-3-one (**86**) [35].

### 4.4. Thiophenes

**Figure 5:**
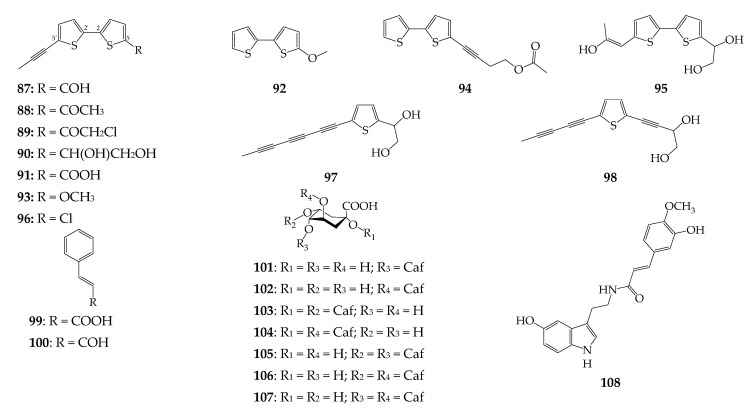
Thiophenes **87**–**98** and hydroxycinnamates **99**–**108**. Caf–caffeoyl.

Twelve thiophenes (**87**–**98**) have been isolated from the roots of *R. uniflorum*, including monomers (**97**, **98**) and dimeric derivatives of 2,2′-dithiophene (**87**–**96**) (Figure 5). Typical thiophenes of *R. uniflorum* are derivatives of 5′-(1-propynyl)-2,2′-dithiophene, with various substituents at position C_5_, such as arctinal (**87**) [17,41], arctinone b (**88**) [17,41,42], and arctic acid (**91**) [17,25,40]. Two chlorinated thiophenes, 7-chloroarctinone b (**89**) [41,42] and rhapontiynethiophene A (**96**) [42], have been isolated from the roots of Chinese origin.

### 4.5. Hydroxycinnamates

Cinnamic acid (**99**) and cinnamaldehyde (**100**) have been found in the *R. uniflorum* flowers [9], while seven caffeoylquinic acids (**101**–**107**) were found to be components of the herb and seeds (Figure 5) [29,30,43]. Feruloyl serotonin (**108**) was isolated from the seeds of *R. uniflorum* [29] and was previously found in *R. carthamoides* [52].

### 4.6. Flavonoids

**Figure 6:**
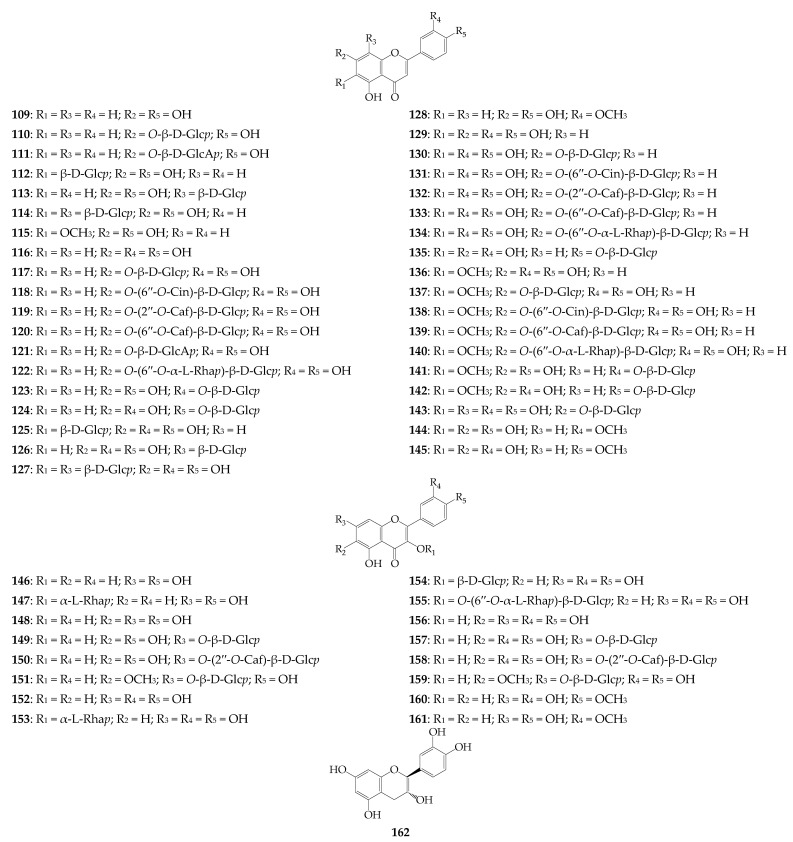
Flavonoids **109**–**162**. Caf–caffeoyl; Cin–cinnamoyl; β-D-Glc*p*–β-D-glucopyranose; β-D-GlcA*p*–β-D-glucuronopyranose; α-L-Rha*p*–α-L-rhamnopyranose.

Flavonoids are the largest group of *R. uniflorum* metabolites containing 53 compounds (**109**–**161**), including 37 flavones (**101**–**145**), 16 flavonols (**146**–**161**) and one catechin (**162**) (Figure 6) [9,16,29,30,33]. Flavone derivatives are present in most *O*- and *C*-glucosides of apigenin (6 compounds), luteolin (12 compounds), 6-hydroxyluteolin (7 compounds), and 6-methoxyluteolin (7 compounds). Glycoside moieties of flavone glycosides contain glucose, glucuronic acid, rutinose, and acylated carbohydrates as cinnamoyl/caffeoyl-glucose attached mainly at C_7_ (18 compounds) and at C_3′_/C_4′_ (5 compounds). Glycosides of kaempferol, 6-hydroxykaempferol, quercetin, and 6-hydroxy/methoxy-quercetin are the main flavonols of *R. uniflorum*. The general structural patterns are very similar to flavones (carbohydrate nature, 7-*O*-glycosylation), and 3-*O*-glycosides have also been detected. The known data indicate that the greatest flavonoid diversity is specific to leaves, which contain 43 compounds [33], followed by the flowers (15 compounds) [9,30] and seeds (4 compounds) [29].

### 4.7. Lignans

**Figure 7:**
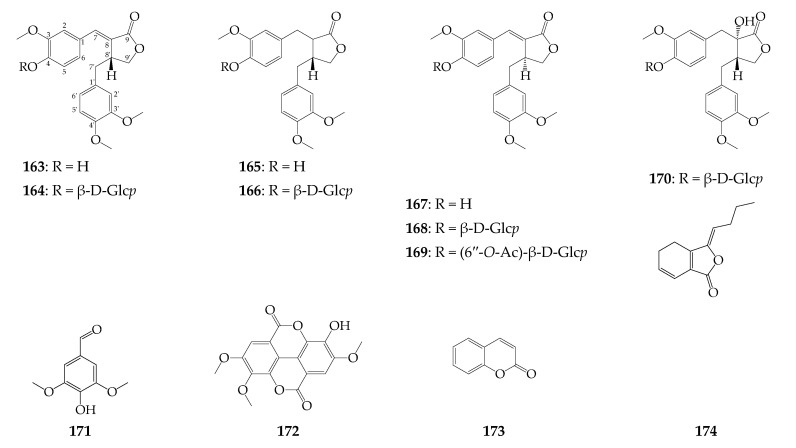
Lignans **163**–**170** and various phenolics **171**–**174**. Ac–acetyl; β-D-Glc*p*–β-D-glucopyranose.

Four lignans have been identified in the herbal part of *R. uniflorum*, which include those widely distributed in Asteraceae arctigenin (**164**), arctiin (**165**) [9], hemislin B (**162**), hemislin B O-glucoside (**163**) [30], found only in *Hemistepta lyrata* (Bunge) Bunge (Asteraceae) (Figure 7) [52]. Later, carthamogenin (**166**) and carthamoside (**167**), which are isomeric to **162** and **163** in the α-position of hydrogen at C_8′_ [53], were isolated from the seeds of *R. uniflorum* together with the acetyl ester of **167** and tracheloside (**169**) [29].

### 4.8. Other Compounds

Among other phenolic components, catechin (**171**) and 3,3′,4-tri-O-methyl-ellagic acid (**172**) in the roots [13] and 3,5-dimethoxy-4-hydroxybanzaldehyde (**170**), coumarin (**173**), and ligustilide (**174**) in the flowers have been identified in *R. uniflorum* [9]. The presence of 13 amino acids (**175**–**187**), including essential amino acids, was found in *R. uniflorum* organs [44]. The main components of the free amino acids were alanine and glycine, while lysine and valine dominated among the bound amino acids. 3′-Deoxyadenosine (cordycepin, **188**) and nicotinamide (**193**) were detected in the flowers [9], and some vitamins (**189**–**192**, **194**, **195**) have been quantified in the herb and roots of *R. uniflorum* [45]. Additionally, four alkanes (**196**–**199**) and fatty acids (**200**–**217**) have been described as components of the whole plant [35]. The main components of the lipid fraction of *R. uniflorum* herb are linolenic acid (19.6%), palmitic acid (18.0%), and linoleic acid (13.4%). Root lipids of *R. uniflorum* are similar to the herb profile; however, the highest content was noted for linoleic acid (41.2%) and lower for palmitic acid (1.8%) and linolenic acid (8.3%). There is also information about essential oil composition in the flowers [54] and roots of *R. uniflorum* [55], including free carbohydrates (**218**–**225**) and polysaccharides [46].

## 5. Chromatographic Analysis of *R. uniflorum*

Despite the widespread use of *R. uniflorum* as a medicinal plant, only few methods for the quantitative analysis of this plant material using liquid chromatography are known (Table 3). To separate the main ecdysteroids of the herb and roots of *R. uniflorum* (**28**, **25, 41**, **50**, **53**), six variants of high performance liquid chromatography analysis on reversed-phase sorbents have been proposed, i.e., using the columns Ultrasphere ODS [7], Zorbax ODS [28], ProntoSIL 120-5 C18 [56], YMC-Pack C18 [57], GLC Mastro C18 [43], and Waters Acquity UPLC HSS T3 C18 [9] with 100–250-mm length [7,9,28] or 60-mm microcolumns [56]. Mixtures of methanol, acetonitrile, water, perchlorate buffer, and formic acid have been used as eluents to achieve separation in isocratic and gradient modes. The total duration of the analysis varied from 15 to 70 min. Analysis of the dominant components of *R. uniflorum* flowers has also been performed under reversed phase HPLC conditions using a mixture of phosphoric acid and acetonitrile [57]. The chosen analysis conditions allowed separation of six compounds, including **28**, **109**, **116**, **128**, **147**, and **163**.

According to the quantitative analysis of *R. uniflorum*, the content of individual compounds in different organs may vary (Table 4). The concentration of the dominant ecdysteroid 20-hydroxyecdysone (**28**) in raw materials collected in Russia was 0.02–1.06% [28,56]. Plants growing in China are characterized by a higher content of **28** in the leaves (up to 1.35%) than in the roots (0.45%) [7,57]. The level of other ecdysteroids (**25**, **41**, **50**, and **53**) was characterized as trace. The concentration of the basic phenolic compounds in *R. uniflorum* flowers varied from 0.03–0.05% for **128** to 0.42–2.26% for **163** [57].

## 6. Bioactivities

The known literature data on bioactivity of *R. uniflorum* are primarily related to the preparation of plant roots in the form of extracts and decoctions, as well as the bioactivity of the leaf, herb, and flower extracts (Table 5). 

### 6.1. Anti-Inflammatory Activity

The study of the anti-inflammatory mechanisms of *R. uniflorum* roots and flowers demonstrated their effectiveness in in vitro and in vivo studies [8,9,16,19,58]. Ethanol extract of *R. uniflorum* roots significantly inhibited the secretion of nitric oxide (NO) and inflammatory cytokines in the culture of RAW 264.7 mouse macrophages and peritoneal macrophages without the manifestation of cytotoxicity [58]. The extract significantly suppressed the expression of inducible NO synthase (iNOS) and cyclooxygenase 2 while simultaneously inducing the expression of heme oxygenase 1 [58]. The inhibition of phosphorylation and degradation of the IκBα factor led to the prevention of nuclear translocation of the NF-κB transcription factor, which, in turn, controls the expression of immune response, apoptosis, and cell cycle genes. A pronounced ability of the *R. uniflorum* root extract to suppress mitogen-activated protein kinases (MAPKs), such as ERK1/2, p38, and JNK, was revealed in a culture of lipopolysaccharide (LPS)-stimulated macrophages [8]. The lipophilic components of the hexane and chloroform fractions of *R. uniflorum* had a greater inhibitory effect on NO production in a culture of LPS-stimulated macrophages and suppressed the transcription of the iNOS messenger RNA [8]. The butanol and ethyl acetate fractions reduced the synthesis of prostaglandin PGE2, while the hexane and ethyl acetate fractions led to the suppression of interleukin-1β [8]. Overall, these facts demonstrate the effectiveness of the *R. uniflorum* root extract as an anti-inflammatory agent acting through the activation of NF-κB and MAPK signaling pathways. Investigation of the anti-inflammatory activity of the *R. uniflorum* flower extract demonstrated its facilitating potential after doxorubicin-initiated cardiotoxicity of embryonic rat cardiomyocytes H9c2 [16]. In in vivo experiments, *R. uniflorum* flower extract prevented LPS-induced pathological alterations of lung bronchoalveolar lavage fluid (BALF) [9]. Downregulation of F4/80 antigen expression in lungs and suppression of LPS-induced elevations in BALF and lung tissue levels of myeloperoxidase were observed with the simultaneous reduction of expression of proteins p-p38, p-JNK, p-ERK (mitogen-activated protein kinase signaling pathway), TLR4, Myd88, p-IκB, and p-p65 (Toll-like receptor 4 and NF-κB signaling pathway) [9]. The abovementioned results indicated that the *R. uniflorum* flower extract ameliorated LPS-induced acute lung injury by suppressing the inflammatory response and enhancing antioxidant capacity.

### 6.2. Antitumor Activity

The root extracts of *R. uniflorum* in in vitro studies reduced the proliferation of AGS human gastric adenocarcinoma cells [59], SCC 15 oral cancer cells [60], and human lung adenocarcinoma cells A549 and H1299 tumor cells [61]. The extracts inhibited messenger RNA (mRNA) and expressed transcription factors protein C-ets-1 (ETS1), and peroxiredoxin 1 (Prx1) resulted in the suppression the growth and proliferation of SCC 15 cells [60]. Animal experiments with H_22_ hepatoma cells demonstrated reduction of transplanted tumor grow caused by reducing DNA fragmentation and microvascular density and worsening the expression of signaling proteins, such as vascular endothelial growth factors (VEGF) and hypoxia-inducible factor 1α (HIF-1α), indicating an antiangiogenic and proapoptotic effect on H_22_ cells [62]. Root ethyl acetate extract affected the growth of SCC15 epidermoid carcinoma cells, reducing their viability and inducing their apoptosis. Treatment of cells with this fraction promoted the expression of messenger RNA and E-cadherin, while reducing the expression of peroxiredoxin 1, vimentin, and the SNAI1 protein influenced the program of the epithelial-mesenchymal transition, significantly reducing tumor growth [63]. The aqueous extract of *R. uniflorum* roots (100–400 mg/kg) slowed tumor growth by 27–38% in mice with transplanted H22 tumors, improving the immune system and antioxidant status of the organism [64]. 

### 6.3. Immune-Stimulating Activity

The immunostimulatory effect of the *R. uniflorum* root extract has been described for the experimental immune suppressions caused by azathioprine, owing to the increasing activity of the cellular, humoral, and macrophage components of the body′s immune system [65]. The extract from the leaves of *R. uniflorum* is an effective immune stimulant in cyclophosphamide-induced immunodeficiency [66].

### 6.4. Nervous System Effects

A study on the anti-anxiety effect of *R. uniflorum* showed that animals treated with dry root extract (200–300 mg/kg) had higher overall locomotor activity compared to control animals. Administration of the *R. uniflorum* extract had a pronounced anti-anxiety effect under conditions of unpunished behavior. An increase in exploratory activity and a decrease in the feeling of fear and anxiety in animals was explained by a decrease in their level of emotionality [67]. The administration of the extract stimulated cognitive functions, accelerated the development of conditioned reflexes, and ensured the long-term preservation of memory. The use of the *R. uniflorum* root extract in mice with galactose-induced aging contributed to the prevention of mitochondrial degeneration, increased the level of succinate dehydrogenase and superoxide dismutase in brain tissues, and decreased the level of MDA, monoamine oxidase, and lactate dehydrogenase activity [68]. Finally, it led to a decrease in the concentration of lipoperoxides and lipofuscin in brain tissues, positively affecting the learning and memory processes [69]. The leaf extract of *R. uniflorum* (50–200 mg/kg) resulted in the adaptation of animals to unfamiliar conditions, an increase in orienting-exploratory activity, and the formation of a conditioned reflex with positive reinforcement, which has generally indicated a pronounced anti-anxiety effect [70]. After 30 min hypobaric hypoxia and 3 h reoxygenation, the use of *R. uniflorum* leaf extract (100 mg/kg) limited the formation of pyknotic neurons, sharply hypochromic neurons, and “shadow cells” in the cortex of cerebral hemispheres, indicating a neuroprotective effect during hypoxia/reoxygenation [71].

### 6.5. Stress-Protective Activity

In models of 18 h immobilization stress and psycho-emotional stress, it was found that extracts from the herb and roots of *R. uniflorum* (100 mg/kg) had a pronounced stress-protective effect, reducing the involution of immunocompetent organs (adrenals, thymus, spleen), delaying the development of deep destruction of the gastric mucosa, reducing the level of MDA, and increasing the concentration of reduced glutathione and the activity of catalase and superoxide dismutase [67]. After administration of *R. uniflorum* extracts, there was a decrease in blood concentration of adrenaline, norepinephrine, adrenocorticotropic hormone, corticosterone, and aldosterone [72]. The positive effect of extracts is due to the limitation of hyperactivation of sympathetic–adrenal and hypothalamic–pituitary–adrenal stress-realizing systems.

### 6.6. Actoprotective and Anabolic Activity

Administration of the *R. uniflorum* root extract (100 mg/kg) led to an increase in overall physical endurance in experimental animals, which affected the increase in working capacity, improved energy supply of working tissues, and increased ATP content in skeletal muscles [68]. A decrease in the severity of metabolic acidosis and the intensity of free radical processes also prolonged the possibility of performing physical work. An increase in the animal body weight, up to 16% compared with the control after application of the *R. uniflorum* root extract (100 mg/kg), occurred owing to an increase in the skeletal muscle mass [67]. An increase in the muscle protein synthesis and DNA and RNA concentrations was observed without a noticeable effect on blood glucose and somatotropic hormone levels, which indicated an anabolic effect of the *R. uniflorum* root extract.

### 6.7. Antihypoxic and Anti-Ischemic Activity

Dry extracts of *R. uniflorum* (50–200 mg/kg) demonstrated pronounced antihypoxic effect, while the effectiveness of root extract was higher in models of hypercapnic and hemic hypoxia, and the herb extract was more effective in histotoxic hypoxia [67]. Intragastric administration of *R. uniflorum* leaf extract (50–200 mg/kg, 14 days) before bilateral carotid artery occlusion led to a decrease in the total mortality of experimental animals, a decrease in neurological deficit, and a decrease in the severity of cerebral edema [73].

### 6.8. Hepatoprotective Activity

Root ethanol extract of *R. uniflorum* increased cell viability at H_2_O_2_-induced liver cell damage in in vitro models [74,75]. Pre-treatment of mice with an aqueous *R. uniflorum* root extract attenuated CCl_4_-induced liver damage, decreased the activity of alanine aminotransferase and aspartate aminotransferase in serum, reduced the concentration of hydroperoxides and malondialdehyde in the liver, increased the level of catalase, glutathione peroxidase, and superoxide dismutase, and reduced glutathione [76]. A decrease in the activity of Na^+^-K^+^-ATPase and Ca^2+^-Mg^2+^-ATPase in liver mitochondria and a decrease in the hepatocyte DNA damage indicated a pronounced hepatoprotective effect of the extract on the function of the damaged organ.

### 6.9. Anti-Aterosclerotic and Hypolypidemic Activity

In a hypercholesterol diet model in birds, the *R. uniflorum* root extract was found to reduce the incidence and severity of atherosclerotic vascular lesions while protecting the ultra-microstructural integrity of cells [77]. The ethanol *R. uniflorum* root extract reduced the levels of triglycerides and the low- and high-density lipoproteins in the blood of mice with experimental hyperlipidemia and prevented lipid accumulation in hepatocytes [78].

### 6.10. Other Activities

Peroxisome activator-activated receptors (PPARs) are a group of nuclear receptors that play an essential role in the regulation of metabolism. Gamma-type receptors (PPARγ) are expressed in all tissues of the body and are a therapeutic target for the treatment of obesity, diabetes, cancer, and other diseases. The *R. uniflorum* root extract, as well as its component 7-chloroarctinone b (**89**), inhibited the rosiglitazone-induced transcriptional activity of PPARγ [79]. Plasmon resonance indicated that **89** binds to PPARγ receptors, blocking the ability of PPARγ agonists to interact with the ligand-binding domains of the receptors (PPARγ-LBD). The ability of **89** to inhibit hormonal and rosiglitazone-induced adipocyte differentiation was confirmed using the Gal4/UAS model and two hybrid yeast methods, indicating its potential efficacy for the treatment of metabolic diseases.

There is also evidence that the aqueous *R. uniflorum* root extract has an antioxidant and membrane-stabilizing activity [43,80,81], an antibacterial effect against *Gardnerella vaginalis* [82], a moderate diuretic effect [58], and a pancreatic α-amylase-inhibiting potential [29].

## 7. Toxicity

The study of acute toxicity of *R. uniflorum* dry extracts from the herb and roots at doses of 3.5–10 g/kg demonstrated no death of animals after intragastric administration [83]. After intraperitoneal administration, the LD_50_ values were 5.8 (herb extract) and 9.5 g/kg (root extract). Long-term administration of the extracts had no negative effect on the morpho-functional parameters of the central nervous, cardiovascular, and urinary systems, organs of the gastrointestinal tract, metabolism, peripheral blood parameters, and the hemostasis system of laboratory animals [83]. Application of the extract as single injection at doses of 100 and 1000 mg/kg did not have local irritating or mutagenic effects. These results indicate that *R. uniflorum* extracts belong to the practically non-toxic group.

## 8. Conclusions

This review summarizes the scientific literature concerning the chemical composition, methods of analysis, and biological activity of traditional medicine *Rhaponticum uniflorum*. The presented data indicate a good degree of knowledge of the metabolites of the roots and herb of *R. uniflorum*. Of particular interest are the anti-inflammatory components of *R. uniflorum*, such as sesquiterpenes [84], ecdysteroids [85], triterpenes [86], thiophenes [87], and flavonoids [88]. Owing to the confirmed presence of these compounds in the plant, we understand its ethnopharmacological use as an anti-inflammatory agent. Despite promising information on the chemical and pharmacological composition of *R. uniflorum* and its extracts, biological studies of individual compounds are still insufficient. We note a lack of studies on metabolites (e.g., sesquiterpenes, triterpenes, and thiophenes) in aboveground organs. The composition of phenolic compounds of the whole plant has not been fully studied to date. Carbohydrates remain an unexplored class of compounds for *R. uniflorum* and the genus *Rhaponticum* in general. It is necessary to expand our knowledge about the organ-specific distribution of substances in the plant, as well as the influence of the environmental conditions of *R. uniflorum* growth on its chemical profile. Owing to the current level of scientific interest in *R. uniflorum* and its extracts, new data on the pharmacological efficacy of pure compounds in various pathologies should be expected in the near future. Therefore, we believe that this review is a starting point for future research on the health benefits of consuming products containing *R. uniflorum*, especially modern dosage forms (e.g., nanoformulations), which will contribute to a wider inclusion of this natural component in new pharmacological products.

## 9. Patents

Available patent information suggests that *R. uniflorum* extracts were registered as components of complex antihypoxic and adaptogenic remedy [89], cosmetic composition with a purpose of lipometabolism promoter [90], soy sauce [91], and granulated insecticide [92], as well as an independent medicine with stress-protective [93] or anxiolytic activity [94].

## Figures and Tables

**Table 3:** HPLC analysis conditions used for the separation of selected *R. uniflorum* metabolites.

Compounds	Column	Elution Mode (I—Isocratic; G—Gradient), Eluents, Gradient Programm; Flow Rate (ν)	Column Temperature (T), Detector ^1^ (D), Analysis Duration (t)	Ref.
**28, 50, 53**	Ultrasphere ODS (250 × 4.6 mm, 5 μm; Hichrom Ltd., Lutterworth, UK)	I; MeOH-H_2_O 40:60; ν 1.5 mL/min	T 20°C; D: UV (λ 242 nm); t 15 min	[7]
**25, 28, 41**	Zorbax ODS (250 × 4.6 m, 5 μm; Agilent Technologies, Santa-Clara, CA, USA)	I; MeCN-H_2_O 20:80; ν 2 mL/min	T 55 °C; D: UV; t 20 min	[28]
**25, 28, 41, 53**	ProntoSIL 120-5 C18 AQ (60 × 1 mm, 1 μm; Knauer, Berlin, Germany)	G; A: 4.1 M LiClO_4_-0.1 M HClO_4_ 5:95, B: MeCN; 0–15 min 5–58% B; ν 0.15 mL/min	T 35 °C; D: UV (λ 248 nm); t 15 min	[56]
**28, 109, 116, 128, 147, 163**	YMC-Pack C18 (250 × 4.6 mm, 5 μm; YMC Co. Ltd., Kyoto, Japan)	G; A: 0.2% H_3_PO_4_, B: MeCN; 0–15 min 20–25% B, 15–50 min 25–40% B; ν 0.8 mL/min	T 35 °C; D: UV (λ 254 nm); t 50 min	[57]
**28, 38, 101–107, 111, 121**	GLC Mastro C18 (150 × 2.1 mm, 3 μm; Shimadzu, Kyoto, Japan)	G; A: 0.5% HCOOH in water, B: 0.5% HCOOH in MeCN; 0–2 min 5–6% B, 2–9 min 6–11% B, 9–15 min 11–25% B, 15–20 min 25–55% B, 20–25 min 55–5% B	T 35 °C; D: PDA (λ 254 nm), MS; t 25 min	[43]
**2, 16, 57, 99, 100, 102, 106, 113, 153–155, 160, 164, 165, 170, 173, 174, 188, 193, 217**	Waters Acquity UPLC HSS T3 C18 (100 × 2.1 mm, 1.8 μm)	G; A: MeCN, B:0.1% HCOOH; 0–10 min 100% B, 10–20 min 100–70% B, 10–25 min 70–60% B, 25–30 min 60–50% B, 30–40 min 50–30% B, 40–45 min 30–0% B, 45–60 min 0% B, 60–60.1 min 0–100% B, 60.1–70min 100% B; ν 0.2 mL/min	T 30 °C; D: DAD (λ 254 nm), MS; t 70 min	[9]

^1^ Detectors: DAD–diode array; MS–mass spectrometric; PDA–photodiode array; UV–ultraviolet.

**Table 4:** Content of selected metabolites in *R. uniflorum* organs, % of dry plant weight.

Origin	Compound									
	25	28	41	50	53	109	116	128	147	163
Roots										
China [7]		0.12–0.45		0.01–0.06	0.01–0.07					
Russia [28,56]	Tr.–0.02	0.09–0.85	Tr.	0.16						
Flowes										
China [7]		0.78		0.02	Tr.					
Russia [28]		0.03								
Leaves										
China [7,41]		0.27–1.35		Tr.–0.09	Tr.	0.08–0.24	0.19–0.60	0.03–0.05	0.66–1.26	0.42–2.26
Russia [28]	Tr.–0.06	0.02–0.85	Tr.–0.02							
Stems										
China [7]		0.62		0.05	0.02					
Russia [28]	Tr.	0.03–0.47	Tr.							
Herb										
Russia [56]	0.24	1.06		0.10	0.21					

Tr.—trace content.

**Table 5:** Bioactivity data of *R. uniflorum*.

Extract, Compound	Assay, Model	Dose ^a^	Positive Control	Result ^b^	Ref.
Anti-inflammatory activity					
In vitro study					
Roots ethanol extract	LPS-stimulation of murine macrophage RAW 264.7 cells	10–100 μg/mL	Dexamethasone (10 μg/mL)	Inhibition NO, TNF-α, IL-6, IL-1β, iNOS, COX-2, HO-1, NF-κB, phospho-IκBα, IκBα, ERK1/2, p38, JNK	[58]
Roots hexane, chloroform, ethyl acetate, butanol, water extracts	LPS-stimulation of murine macrophage RAW 264.7 cells	5–100 μg/mL	N^G^-monomethyl-L- arginine monoacetate (10 μM)	Inhibition NO, PGE2, IL-1β, IL-6, iNOS	[8]
Flower ethanol extract	Doxorubicin-initiated cardiotoxicity of embryonic rat cardiomyocytes H9c2	12.5–800 μg/mL	Dexrazoxane (7.5 μg/mL)	Inhibition ROS, Bax, cleaved-caspase-3, cleaved-caspase-9, cleaved-PARP, NF-κB	[16]
In vivo study					
Flower ethanol extract	Oropharyngeal aspirational LPS induced acute lung injury of male BALB/c mice	100–400 mg/kg	Dexamethasone (5 mg/kg)	Inhibition TNF-α, IL-6, NO, p-p38, p-JNK, p-ERK, TLR4, Myd88, p-IκB, p-p65, Keap1; stimulation Nrf2, HO-1, NQO1	[9]
Antitumor activity					
In vitro study					
Root ethanol extract	AGS human gastric adenocarcinoma cell	50–150 μg/mL	5-Fluorouracil (5 mg/kg)	Inhibition of tumor cells grow	[59]
Roots ethyl acetate extract	Cell carcinoma cell line SCC15	50 μg/mL	5-Fluorouracil (5 μg/mL)	Inhibition tumor grow, ETS1, Prx1	[60]
Root methylene chloride, ethyl acetate, butanol extracts	Human lung adenocarcinoma cells A549 and H1299	10–500 μg/mL	5-Fluorouracil (5 mg/kg)	Inhibition of tumor cells grow	[61]
In vivo study					
Roots ethanol extract	Mice bearing H_22_ hepatoma cells	100–400 mg/kg p.o.	5-Fluorouracil (5 mg/kg)	Anti-angiogenic and pro-apoptotic effects against H22 hepatoma cells	[62]
Roots ethyl acetate extract	Human OSCC cell line SCC15	12.5–100 μg/mL	5-Fluorouracil (5 mg/kg)	Induction of apoptosis; suppression of cell invasion and migration; inhibition Prx1, vimentin, Snail	[63]
Roots water extract	Mice bearing H_22_ hepatoma cells	100–400 mg/kg p.o.	5-Fluorouracil (5 mg/kg)	Inhibition tumor grow, TNF-α	[64]
Immune-stimulating activity: in vivo study					
Roots ethanol extract	Erythrocyte immune function of rats	3–15 mg/kg; i.p.	-	Enhancement of erythrocyte immune function	[65]
Leaf ethanol extract	Cyclophosphamide-induced immunodeficiency of CBA×C57Bl/6 mice	100 mg/kg; i.p.	Echinacea extract (200 mg/kg)	Increasing of the cellular, humoral, and macrophage immunity	[66]
Nervous system effects: in vivo study					
Roots ethanol extract	Elevated plus maze test and dark/light chamber of Wistar rats	100–300 mg/kg; p.o.	*Rhaponticum carthamoides* extract (100 mg/kg)	Anti-anxiety effect	[67]
Roots ethanol extract	D-galactose-induced aging of mice	20–100 mg/kg; p.o.	-	Anti-aging effect	[68]
Roots ethanol extract	Passive avoidance test of mice	20–100 mg/kg; p.o.	-	Improving memory impairment	[69]
Leaf ethanol extract	Passive avoidance test of mice	50–200 mg/kg; p.o.	*Rhaponticum carthamoides* extract (100 mg/kg)	Anxiolytic effect	[70]
Leaf ethanol extract	Hypoxia/reoxygenation of Wistar rats	100–200 mg/kg; p.o.	*Rhaponticum carthamoides* extract (100 mg/kg)	Neuroprotective effect	[71]
Stress-protective activity: in vivo study					
Roots ethanol extract	Immobilization stress and psycho-emotional stress tests of Wistar rats	100–300 mg/kg; p.o.	*Rhaponticum carthamoides* extract (100 mg/kg)	Stress-protective effect	[67,72]
Actoprotective and anabolic activity: in vivo study					
Roots ethanol extract	Physical endurance test of Wistar rats	100–300 mg/kg; p.o.	*Rhaponticum carthamoides* extract (100 mg/kg)	Increasing of overall physical endurance, working capacity, ATP in muscles, skeletal muscle mass; decrease metabolic acidosis	[67,68]
Antihypoxic and anti-ischemic activity: in vivo study					
Roots ethanol extract	Hypercapnic, hemic, histotoxic hypoxia of Wistar rats	50–200 mg/kg; p.o.	*Rhaponticum carthamoides* extract (100 mg/kg)	Antihypoxic effect	[67]
Leaf ethanol extract	Bilateral carotid artery occlusion of Wistar rats	50–200 mg/kg; p.o.	*Rhaponticum carthamoides* extract (100 mg/kg)	Decrease mortality, neurological deficit, severity of cerebral edema	[73]
Hepatoprotective activity					
In vitro study					
Root ethanol extract	H_2_O_2_-induced liver cells damage	12.5–400 μg/mL	-	Icreasing cell viability; reduction LDH, ALT, AST, MDA; increasing GSH	[74]
Root ethanol extract	H_2_O_2_-induced HepG2 cells damage	25–400 μg/mL	-	Icreasing cell viability, SOD, GSH; reduction LDH, ALT, AST, MDA, caspase-3, 8, 9, cytoplasmic cytochrome C, p-JNK, nuclear NF-κB	[75]
In vivo study					
Roots water extract	Carbon tetrachloride-induced acute liver injury of mice	50–200 mg/kg; i.p.	Bifendate (10 mg/kg)	Reduction serum ALT, AST, liver level of LOOH, MDA; increasing liver CAT, GSH-Px, SOD, Mn-SOD, Na^+^-K^+^-ATPase and Ca^2+^-Mg^2+^-ATPase; DNA damage of hepatocyte	[76]
Anti-aterosclerotic and hypolypidemic activity: in vivo study					
Root ethanol, water extract	Hypercholesterol diet of mice	100–400 mg/kg; p.o.	-	Decreasing total cholesterol, total glycerides, LDL-C; icreasing HDL-C	[77]
Root ethanol extract	Oleic acid-induced fat accumulation in HepG2 cells	10–500 μg/mL; p.o.	-	Decreasing total cholesterol, total glycerides, LDL-C; icreasing HDL-C	[78]
Inhibition of PPARγ receptors: in vitro study					
Roots ethanol extract; 7-chloroarctinone b	Cell-based transactivation assay	1.18–10 μM	-	Inhibition of rosiglitazone-induced transcriptional activity of PPARγ	[79]
Antioxidant activity: in vitro study					
Root water extract	Total antioxidant activity, hydroxyl radical scavenging, Fe2+-induced lipid peroxidation in liver mitochondria	0–100 μg/mL	Ascorbic acid	Antioxidant activity	[80]
Root butanol extract	Total antioxidant activity, hydroxyl radical scavenging, Fe^2+^-induced lipid peroxidation in liver mitochondria	0–100 μg/mL	Ascorbic acid	Antioxidant activity	[81]
Herb ethanol extract	Radical-scavenging activity against 2,2-diphenyl-1-picrylhydrazyl radicals; 2,2′-azino-bis (3-ethylbenzothiazoline-6-sulfonic acid cation-radicals; superoxide radicals; Fe^2+^-chelating activity	5–1000 μg/mL	Ascorbic acid	Antioxidant activity	[43]
Antibacterial activity: in vitro study					
Root water extract	Inhibition of *Gardnerella vaginalis*	0–20 mg/mL	Ampicillin	Bacterial grow inhibition	[82]
Diuretic activity: in vivo study					
Root water extract	3-Month application of extract solution by Wistar rats	100–500 mg/mL; p.o.	-	Moderarte increase of diuresis	[58]
Antidiabetic activity: in vitro study					
Seed water extract, flavonoids, lignans	Inhibition of pancreatic α-amylase	0–100 μg/mL	Acarbose	Moderarte inhibition of α-amylase	[29]

^a^ p.o.–per os, oraly; i.p.–intraperitonealy. ^b^ ALT–alanine transaminase; AST–aspartate transaminase; Bax–Bcl-2-associated X protein; CAT–catalase; COX-2–cyclooxygenase-2; ERK–extracellular signal-regulated kinase 1/2; ETS1–protein C-ets-1; GSH–glutathione reduced; HDL–high-density lipoprotein; HO-1–heme oxygenase 1; IL-6–interleukin-6; IL-1β–interleukin-1β; iNOS–inducible nitric oxide synthase; IκBα–nuclear factor of kappa light polypeptide gene enhancer in B-cells inhibitor, alpha; JNK–c-Jun N-terminal kinase; Keap1–Kelch-like ECH-associated protein 1; LDH–lactate dehydrogenase; LDL–low-density lipoprotein; LOOH–lipid hydroperoxide; MDA–malondialdehyde; Myd88–myeloid differentiation primary response 88; NF-κB–nuclear factor kappa B; NO–nitric oxide (II); NQO1–NAD(P)H dehydrogenase [quinone] 1; Nrf2–nuclear factor erythroid 2-related factor 2; PARP–poly ADP ribose polymerase; PGE2–prostaglandin E2; Prx1–peroxiredoxin-1; p38–mitogen-activated protein kinase p38; ROS–reactive oxygen species; SOD–superoxide dismutase; TNF-α–tumor necrosis factor-alpha; and TLR4–toll-like receptor 4. “-”–no data.

## Data Availability

Data is contained within the article.
